# The Influence of Heredity upon Deformities and Defects

**Published:** 1888-03

**Authors:** 


### The Influence of Heredity upon Deformities and Defects.

The *British Medical Journal* says that some interesting observations bearing upon intermarriage and the recurrence or exaggeration of hereditary defects were brought before the Clinical Society recently, when Mr. Clement Lucas read a paper on "The Congenital Absence of an Upper Lateral Incisor Tooth as a Forerunner of Hare-lip and Cleft-palate." It is now very generally recognized that a defect from arrest of development in any part is liable to be repeated again and again, in succeeding generations, in spite of the introduction of new blood at each marriage. In illustration of this, Mr. Lucas referred to a former observation of his, that among eighty descendants of a woman who had supernumerary digits, thirty per cent. presented a similar deformity. He also alluded to the case of a woman who married after having been successfully operated upon for cleft-palate and harelip; two of her six children presented a deformity similar to that of the mother. He now wishes us to go a step further, and detect a deformity, as it were, in an initial stage. He brought forward three cases to prove that a tendency to the congenital absence of a lateral incisor tooth might be transmitted as such, or might become the parent of hare-lip or cleft-palate. The absence of this tooth, it was explained, indicated an arrest of development along a particular line, which might in a future generation extend either to the lip or palate. Dr. Goodhart showed at the same meeting five children of one family, all of whom had nystagmus and staggering gait. The parents were first cousins, and there had been a former marriage of cousins on the mother's side, whilst on the father's side several relatives were deaf.

